# Three-port *versus* four-port technique for laparoscopic cholecystectomy: systematic review and meta-analysis

**DOI:** 10.1093/bjsopen/zrac013

**Published:** 2022-03-31

**Authors:** Lawrence Nip, Kin-Seng Tong, Cynthia M. Borg

**Affiliations:** Department of General Surgery, University Hospital Lewisham, London, UK; Department of General Surgery, Queen Alexandra Hospital, Portsmouth, UK; Department of General Surgery, University Hospital Lewisham, London, UK

## Abstract

**Background:**

The four-port laparoscopic technique is the standard approach for cholecystectomy. A three-port technique has been described, but there is no consensus over the outcomes and efficacy of this approach. The aim was to perform a systematic review and meta-analysis to compare the three- and four-port techniques in laparoscopic cholecystectomy for benign diseases of the gallbladder.

**Methods:**

The review was conducted according to a predefined protocol registered on PROSPERO. Two authors independently conducted an electronic database search of CENTRAL, MEDLINE, Embase, CINAHL, WHO International Clinical Trials Registry, and ClinicalTrials.gov. Outcomes are reported as risk ratios (RR), mean difference (m.d.), or standardized mean difference (s.m.d.) with 95 per cent confidence intervals.

**Results:**

Eighteen trials were included with 2085 patients. Length of hospital stay and postoperative analgesia requirement favoured the three-port group (m.d. −0.29, 95 per cent c.i. −0.43 to −0.16 (*P* < 0.001); and s.m.d. −0.68, 95 per cent c.i. −1.03 to −0.33 (*P* < 0.001), respectively). There were no differences in length of procedure or success rate between the two groups (m.d. 0.90, 95 per cent c.i. −3.78 to 5.58 (*P* = 0.71) and RR 0.99, 95 per cent c.i. 0.97 to 1.01 (*P* = 0.17), respectively). There were no differences in adverse events. The overall quality of evidence was low.

**Conclusion:**

The three-port technique for laparoscopic cholecystectomy is an option for appropriately trained surgeons who perform it regularly. However, the decision to use three ports should not be at the expense of safe dissection of Calot’s triangle.

## Teaser

This is a systematic review and meta-analysis of outcomes for the three-port *versus* four-port approach in laparoscopic cholecystectomy. We conclude that the three-port technique can be chosen by experienced surgeons; however, is not necessarily the best technique for more complicated cases.

## Introduction

**Fig. 1:**
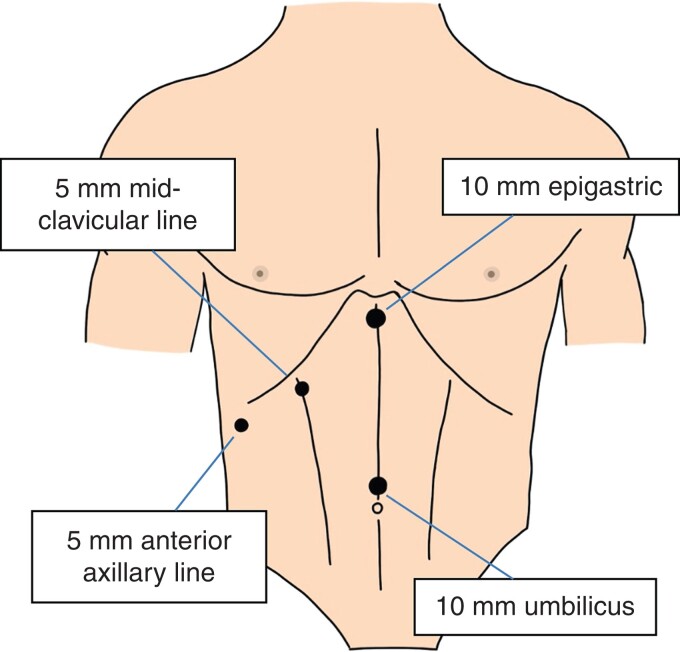
Port placement and sizes of each port for the standard four-port technique

Gallstones are common, with an estimated prevalence of 10 to 15 per cent in the UK adult population^1^. While most people with gallstones remain asymptomatic, around 1 to 2 per cent per year will develop symptoms for which the definitive treatment is cholecystectomy^2^. The four-port technique is currently the standard technique for performing laparoscopic cholecystectomy (*Fig. 1*)^3^.

**Fig. 2:**
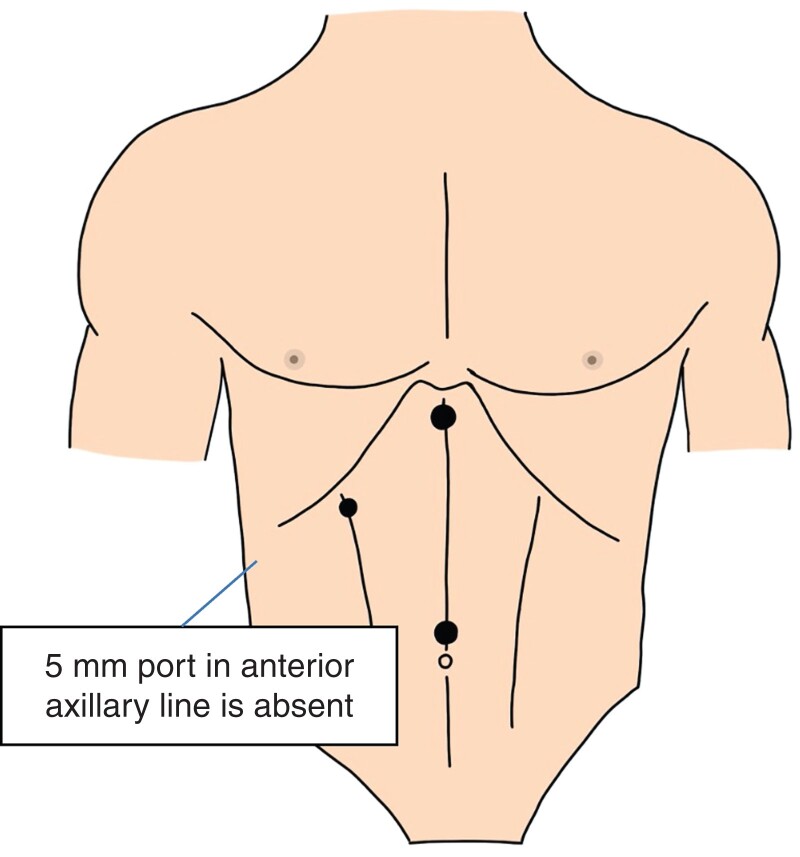
The same equipment is used in the three-port technique but the 5 mm port in the anterior axillary line is absent

Newer techniques exist, including the three-port technique (*Fig. 2*)^4^ using conventional laparoscopic equipment. However, the lateral-most port used for retracting the gallbladder fundus over the surface of the liver is absent. Instead, the gallbladder infundibulum is held via the right upper quadrant port (mid-clavicular line), and this on its own is used to facilitate views of Calot’s triangle.

RCT, randomized controlled trial.

The rationale behind the three-port technique is that good views of Calot’s triangle may still be gained without fundal retraction. With one less incision, tissue trauma is reduced leading to less pain and inflammation. Several early studies demonstrated that the three-port technique was feasible and had comparable outcomes to the four-port technique^5–7^. This is particularly important in an era where single-incision laparoscopic surgery has lost popularity and with few centres having the necessary equipment^8^.

Our scoping literature searches have suggested that the volume of RCTs specifically looking at three-port *versus* four-port laparoscopic cholecystectomy has increased over the last 5 years.

The aim was to perform a comprehensive systematic review and meta-analysis of outcomes to compare the three-port technique to the standard four-port technique in laparoscopic cholecystectomy for benign diseases of the gallbladder.

## Methods

The systematic review was completed according to a predefined protocol, which has been listed on the International Prospective Register of Systematic Reviews (PROSPERO; registration number CRD4202021071813)^9^. The study was completed in adherence with the PRISMA statement (*Appendix S1*)^10^.

### Eligibility criteria

Inclusion criteria were full-text RCTs, written in English, of adults aged 18 years or older undergoing laparoscopic cholecystectomy for benign gallbladder disease in the emergency or elective setting. All three- and four-port techniques with any sized trocars for laparoscopic cholecystectomy were included.

Exclusion criteria were observational studies, non-RCT interventional studies, conference abstracts, editorials, expert opinions, case reports, non-English language articles, non-availability of the full text, studies of participants younger than 18 years of age, studies on cadaveric or animal models, and malignant gallbladder disease. In addition, studies using single-incision/single-site (SILS/SSLC) techniques were excluded. However, studies comparing SILS/SSLC *versus* two-port *versus* three-port *versus* four-port (in any combination) were included if the outcomes of the three-port and four-port techniques were reported separately.

### Outcomes

Primary outcomes included length of hospital stay, length of procedure, postoperative analgesia requirement, and success rate—defined as the procedure being completed without the addition of another port or open conversion.

Secondary outcomes included pain score on visual analogue scale (VAS) or numerical rating scale (NRS), bile duct injury, gallbladder perforation, bile/stone spillage, liver bed bleeding, wound infection, mortality, and cosmetic satisfaction on a VAS or NRS.

### Search strategy

Two authors independently searched the articles for inclusion (L.N. and K.S.T.). Any discrepancies in article selection were resolved by mutual discussion. The databases searched were MEDLINE (from 1946 to October 2020), Embase (from 1974 to October 2020), CENTRAL (October 2020, issue 9), CINAHL (from 1981 to October 2020), the WHO International Clinical Trials Registry Platform (ICTRP; October 2020), and ClincialTrials.gov (October 2020). The reference lists of all included full-text articles and previous systematic reviews were screened, in order to identify any further eligible studies. The authors of included articles were contacted, where possible, to obtain non-published information. The SIGLE database was used to search for grey literature.

The following search terms were used: three-port (OR synonyms) AND four-port (OR synonyms) AND laparoscopic cholecystectomy (OR synonyms). A detailed description can be found in *Appendix S2*.

### Data extraction

Data extraction was done manually by two independent authors (L.N. and K.T.)^11^, and included author, year of publication, country, journal, dates of the study, sample size and group sizes, length of follow-up, inclusion and exclusion criteria, patient demographics (age, sex, BMI), trocar size, outcomes, and risk of bias.

### Risk of bias assessment

Bias was assessed using the Cochrane Risk of Bias 2 tool^12^. Each study was graded as low risk, unclear risk, or high risk of bias. The highest risk score from any one domain was used to inform the overall risk. If the highest risk score was ‘unclear risk of bias’ but occurred across multiple domains, it was classed as high risk of bias. Therefore, to be of low risk of bias overall, the trial had to be at low risk of bias across all six domains.

### Statistical analysis

A meta-analysis was performed using RevMan 5.4^13^ for all prespecified outcomes if three or more studies reported the outcome. A random-effects model was used due to anticipated heterogeneity. Results were reported in a Forest plot with 95 per cent confidence intervals.

Heterogeneity was assessed via three means: visual inspection of overlapping confidence intervals; χ^2^ test; and the *I^2^* statistic. To explore potential sources of heterogeneity, a subgroup analysis of studies at low risk of bias *versus* those with unclear and high risk of bias was performed. A subgroup analysis was performed on emergency *versus* electively operated patients and all outcomes were sensitivity tested.

### Quality of evidence

GRADE levels of certainty were used^14^. As included trials were RCTs, the initial quality of evidence started as high but was rated down if there were any concerns regarding risk of bias, inconsistency, indirectness, imprecision, and other biases. Summaries of the effect estimate, and the overall quality of evidence are presented.

## Results

**Fig. 3:**
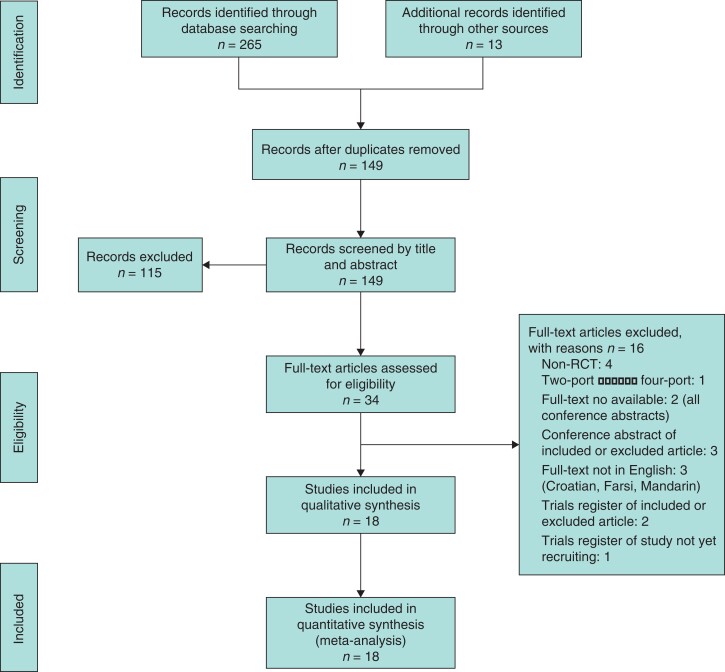
Study flow diagram showing the number of records identified and excluded at each stage

In total, 265 articles were identified from the search strategy with an additional 13 articles identified through searching the ICTRP, ClinicalTrials.gov, the SIGLE grey literature database, and screening the reference lists of all included full-text articles. The flow diagram with the number of and reasons for exclusions at each stage is provided in *Fig. 3*.

Reasons for exclusion of full-text articles are provided.

Eighteen trials were included in our meta-analysis, which randomized 2085 patients into a three-port laparoscopic cholecystectomy group (*n* = 1044) and four-port laparoscopic cholecystectomy group (*n* = 1041). The characteristics of each study can be found in the study characteristics tables (*Tables 1* and *2* and references in *Appendix S3*).

**Table 1:** Baseline characteristics of the included studies

Author	Year	Country	Journal	Study Dates	Sample size
**Agarwal**	2018	India	*International Journal of Medical Research Professionals*	n.r.	40
**Bari**	2019	India	*International Journal of Research in Medical Sciences*	July 2015–March 2017	100
**Cerci**	2007	Turkey	*Hepato-Gastroenterology*	1998–2003	146
**Eroler**	2016	Turkey	*International Journal of Clinical and Experimental Medicine*	n.r.	60
**Gupta**	2005	India	*Tropical Gastroenterology*	January 2004–December 2004	80
**Harsha**	2013	India	*Journal of Medical Society*	September 2010–August 2012	50
**Khorgami**	2014	Iran	*Journal of Investigative Surgery*	June 2011–December 2011	60 (90 with 3 groups)
**Kumar**	2007	Nepal	*Journal of the Society of Laparoendoscopic Surgeons*	August 2004–July 2005	75
**Liu**	2016	China	*International Journal of Clinical and Experimental Medicine*	May 2013–December 2014	216
**Mohamed**	2020	Egypt	*The Egyptian Journal of Surgery*	2018–2019	94
**Moran**	2014	Turkey	*European Journal of Endoscopic and Laparoscopic Surgeons*	February 2009–December 2009	30 (60 with 4 groups)
**Reshie**	2015	India	*International Journal of Advanced Research*	August 2010–September 2014	200
**Shah**	2017	Pakistan	*Rawal Medical Journal*	January 2013–June 2013	60
**Sharma**	2015	India	*JK Science*	n.r.	200
**Singal**	2017	India	*World Journal of Laparoscopic Surgery*	April 2014–March 2015	200
**Singhal**	2019	India	*International Surgery Journal*	September 2018–April 2019	214
**Trichak**	2003	Thailand	*Surgical Endoscopy*	1998–2000	200
**Vejdan**	2020	Iran	*Journal of Surgery and Trauma*	n.r.	60

n.r., not reported.

**Table 2:** Baseline characteristics of the included population

Author	Group sizes	Groups	Follow-up	Mean (s.d.) age (years)	Female (male) sex	Mean (s.d.) BMI (kg/m^2^)	Trocar size
**Agarwal**	20 *versus* 20	Three-port Four-port	n.r.	43.1 44.5	12 (8) 13 (7)	n.r. n.r.	n.r. n.r.
**Bari**	50 *versus* 50	Three-port Four-port	1 month	38 (12) 41 (10)	38 (12) 41 (10)	n.r. n.r.	10–10–5 10–10–5–5
**Cerci**	73 *versus* 73	Three-port Four-port	n.r.	50.08 (12.5) 49.77 (13.6)	54 (19) 55 (18)	29.2 28.7	10–10–5 10–10–5–5
**Eroler**	30 *versus* 30	Three-port Four-port	n.r.	n.r. n.r.	27 (3) 25 (5)	n.r. n.r.	10–10–5 10–10–5–5
**Gupta**	40 *versus* 40	Three-port Four-port	n.r.	26 (11.1) 27 (11.2)	28 (12) 30 (10)	20.2 (6.2) 19.5 (6.1)	5–10–5 10–10–5–5
**Harsha**	25 *versus* 25	Three-port Four-port	1 month	39.10 (13.93) 40.48 (11.04)	17 (8) 21 (4)	24.54 (3.62) 25.13 (2.79)	10–10–5 10–10–5–5
**Khorgami**	30 *versus* 30 (*versus* 30)	Three-port vs four-port (*versus* SILS)	12 months	41.7 (11.2) 41.5 (11.1)	20 (10) 21 (9)	28.6 (4.5) 26.7 (4)	10–5–5 10–5–5–5
**Kumar**	36 *versus* 39	Three-port Four-port	1 month	38.22 (13.67) 39.13 (14.10)	30 (6) 32 (7)	n.r. n.r.	11–10–5 11–10–5–5
**Liu**	110 *versus* 106	Three-port Four-port	3 months	53.2 (12.1) 52.6 (13.2)	63 (47) 66 (40)	23.1 (2.2) 23.7 (2.8)	10–10–5 10–10–5–5
**Mohamed**	45 *versus* 49	Three-port Four-port	1 month	38.26 (13.6) 37.65 (11.69)	36 (9) 44 (5)	n.r. n.r.	10–10–5 10–10–5–5
**Moran**	15 *versus* 15 (*versus* 15)	Three-port *versus* four-port (*versus* SILS *versus* two-port)	n.r.	45.2 (12) 42.8 (8.3)	12 (3) 10 (5)	30.8 (5.6) 30.6 (5.6)	10–10–5 10–10–5–5
**Reshie**	100 *versus* 100	Three-port Four-port	3 months	38.74 (13.38) 39.04 (9.12)	82 (18) 82 (18)	n.r. n.r.	10–10–5 10–10–5–5
**Shah**	30 *versus* 30	Three-port Four-port	7 days	44 (12.9) 44 (12.9)	n.r. n.r.	n.r. n.r.	n.r. n.r.
**Sharma**	100 *versus* 100	Three-port Four-port	n.r.	40.08 (14.64) 50.66 (12.56)	85 (15) 66 (34)	n.r. n.r.	10–10–5 10–10–5–5
**Singal**	100 *versus* 100	Three-port Four-port	3 months	39.33 39.33	n.r. n.r.	n.r. n.r.	10–10–5 10–10–5–5
**Singhal**	110 *versus* 104	Three-port Four-port	1 month	45.4 (6.2) 46.3 (8.6)	102 (8) 97 (7)	n.r. n.r.	10–10–5 10–10–5–5
**Trichak**	100 *versus* 100	Three-port Four-port	n.r.	53.22 (15.31) 53.74 (15.05)	75 (25) 73 (27)	n.r. n.r.	10–5–5 10–5–5–5
**Vejdan**	30 *versus* 30	Three-port Four-port	n.r.	61.10 (4.7) 59.823 (7.8)	25 (5) 24 (6)	27.66 (1.45) 27.57 (1.93)	10–10–5 10–10–5–5

The upper and lower values represent the three and four-port groups, respectively. Nomenclature for trocar size: umbilicus–epigastric–right upper quadrant ± right flank. n.r., not reported; SILS, single incision.

### Risk of bias in included studies

**Fig. 4:**
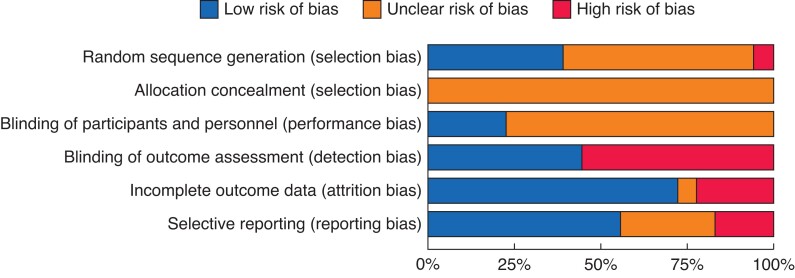
Risk of bias summary

**Fig. 5:**
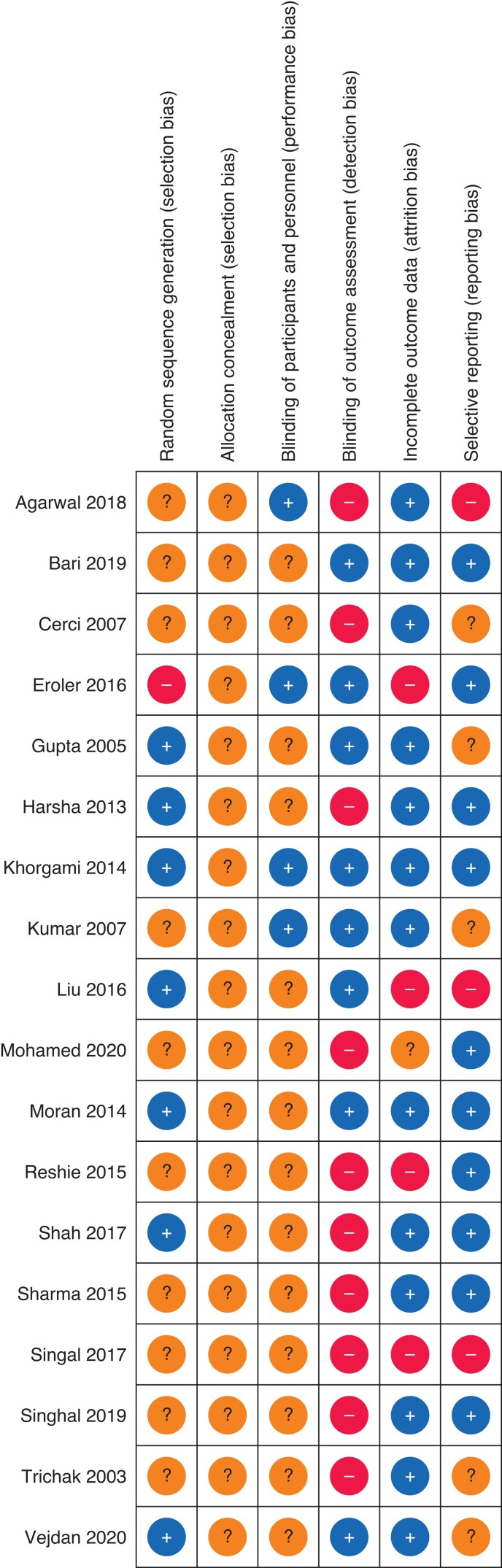
Risk of bias summary

No trials were felt to be at low risk of bias. Twelve studies were felt to be at high risk of bias and six were felt to be at unclear risk of bias (*Figs 4* and *5*). A detailed assessment can be found in *Appendix S4*.

Authors’ judgements regarding each of the six bias domains expressed as the percentage of high risk of bias, unclear risk of bias, and low risk of bias trials.

Authors’ judgements for each risk of bias domain for the included studies.

### Primary outcomes

#### Length of hospital stay

**Fig. 6:**
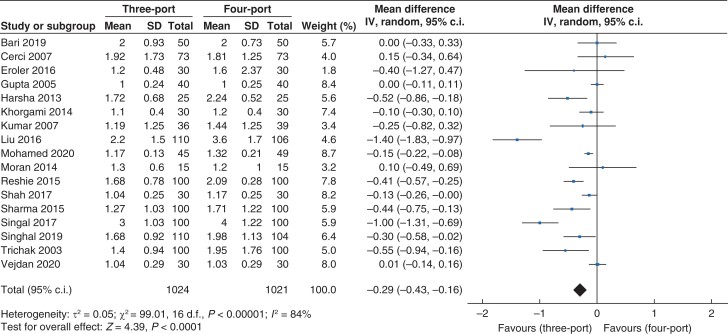
Forest plot for length of hospital stay

Data for length of hospital stay (*Fig. 6*) were reported in 17 trials which recruited 2045 patients (1024 *versus* 1021 in the three- and four-port groups, respectively). The length of hospital stay was lower in the three-port group than in the four-port group (mean difference (m.d.) −0.29, 95 per cent c.i. −0.43 to −0.16; *P* < 0.001). A high level of heterogeneity was found (*I^2^* = 84 per cent, χ^2^ = 99.01; *P* < 0.001).

#### Length of procedure

**Fig. 7:**
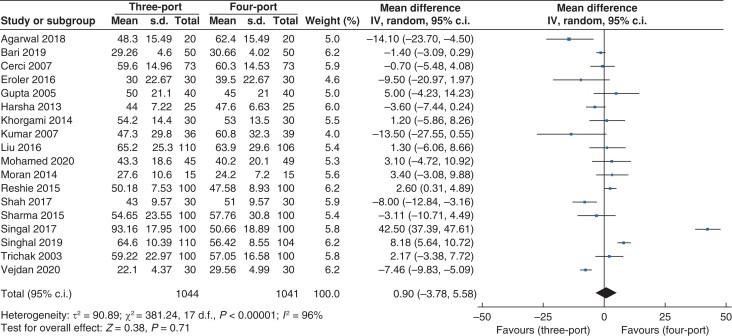
Forest plot for length of procedure

Data for length of procedure (*Fig. 7*) were reported in 18 trials that recruited 2085 patients (*n* = 1044 *versus n* = 1041 in the three- and four-port groups, respectively). There was no statistically significant difference in length of procedure between the three- and four-port groups (m.d. 0.90, 95 per cent c.i. –3.78 to 5.58; *P* = 0.71). A high level of heterogeneity was observed (*I^2^* = 96 per cent, χ^2^ = 381.24; *P* < 0.001).

#### Postoperative analgesia requirement

**Fig. 8:**
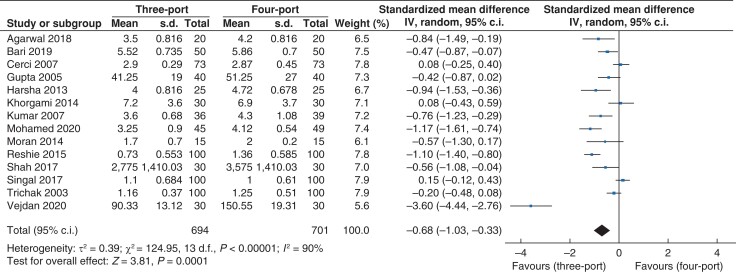
Forest plot for postoperative analgesia requirement

Data for postoperative analgesia requirement (*Fig. 8*) were reported by 14 trials which recruited 1395 patients (*n* = 694 *versus n* = 701 in the three- and four-port groups, respectively). Standardized mean difference (s.m.d.) was used owing to differences in local policy for analgesic regimes in the postoperative period. The postoperative analgesia requirement was lower in the three-port group than the four-port group (s.m.d. −0.68, 95 per cent c.i. −1.03 to −0.33; *P* < 0.001). A high level of heterogeneity was observed (*I^2^* = 90 per cent, χ^2^ = 124.95; *P* < 0.001).

#### Success rate

**Fig. 9:**
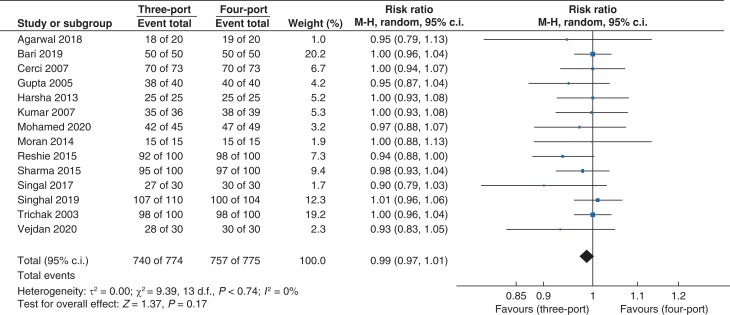
Forest plot for success rate

Success rate was defined as the ability to complete the procedure without the addition of an extra port or open conversion. Data for this outcome (*Fig. 9*) were reported in 14 trials that recruited 1549 patients (*n* = 774 *versus n* = 775 in the three- and four-port groups, respectively). There was no statistically significant difference in the success rate between the three- and four-port groups (risk ratio (RR) 0.99, 95 per cent c.i. 0.97 to 1.01; *P* = 0.17). A low level of heterogeneity was observed (*I^2^* = 0 per cent, χ^2^ = 9.39; *P* = 0.74).

### Secondary outcomes

#### Pain score

**Fig. 10:**
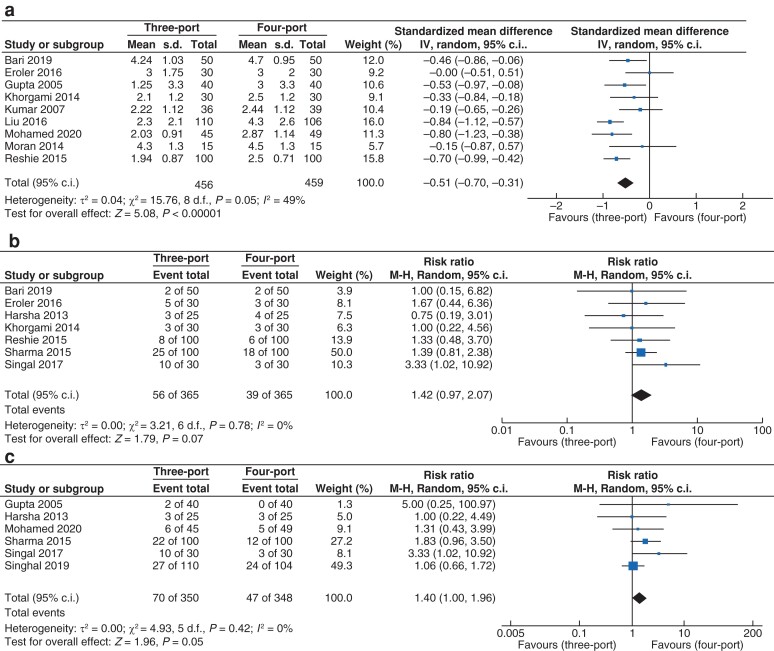

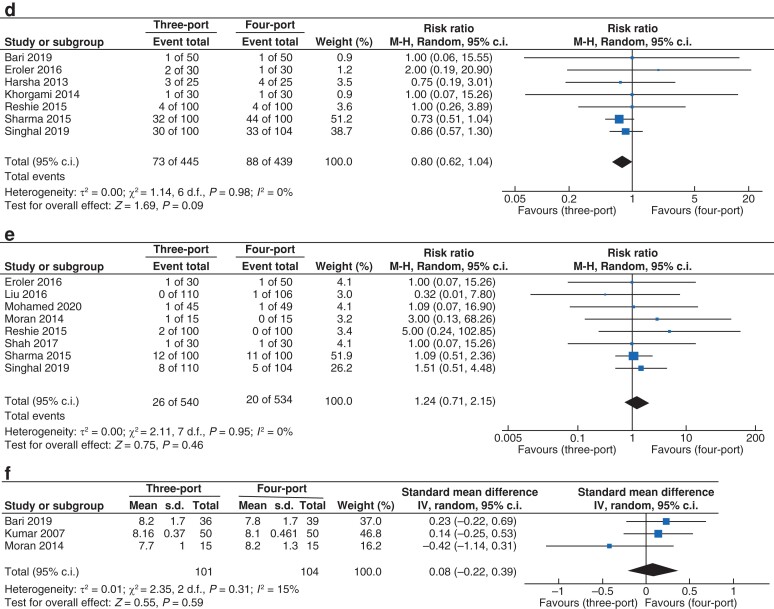
Forest plots for the following secondary outcomes

Data for pain score (*Fig. 10a*) were reported by nine trials that recruited 915 patients (*n* = 456 *versus n* = 459 in the three- and four-port groups, respectively). Meta-analysis was performed on studies reporting a pain score for participants at 24 hours postoperatively. Standardized mean difference was used as there were differences in the pain scales used by different institutions. The pain score at 24 hours was lower in the three-port group *versus* the four-port group (s.m.d. −0.51, 95 per cent c.i. −0.70 to −0.31; *P* < 0.001). A moderate level of heterogeneity was observed (*I^2^* = 49 per cent, χ^2^ = 15.76; *P* = 0.05).

**a** Pain score at 24 h; **b** gallbladder perforation; **c** spillage of biliary contents; **d** liver bed bleeding; **e** wound infection; **f** cosmetic satisfaction at 7 days.

#### Gallbladder perforation

Data for gallbladder perforation (*Fig. 10b*) were reported by seven trials that recruited 730 patients (*n* = 365 *versus n* = 365 in the three- and four-port groups, respectively). There was no statistically significant difference in the rate of gallbladder perforation between the three- and four-port groups (RR 1.42, 95 per cent c.i. 0.97 to 2.07; *P* = 0.07). A low level of heterogeneity was observed (*I^2^* = 0 per cent, χ^2^ = 3.21; *P* = 0.78).

#### Spillage of biliary contents

Data for spillage of biliary contents (*Fig. 10c*) were reported by six trials that recruited 698 patients (*n* = 350 *versus n* = 348 in the three- and four-port groups, respectively). There was no statistically significant difference in the rate of spillage of biliary contents between the three- and four-port groups (RR 1.40, 95 per cent c.i. 1.00 to 1.97; *P* = 0.05). A low level of heterogeneity was observed (*I^2^* = 0 per cent, χ^2^ = 4.93; *P* = 0.42).

#### Liver bed bleeding

Data for liver bed bleeding (*Fig. 10d*) were reported by seven trials that recruited 884 patients (*n* = 445 *versus n* = 439 in the three- and four-port groups, respectively). There was no statistically significant difference in the rate of liver bed bleeding between the three- and four-port groups (RR 0.80, 95 per cent c.i. 0.62–1.04; *P* = 0.09). A low level of heterogeneity was observed (*I^2^* = 0 per cent, χ^2^ = 1.14; *P* = 0.98).

#### Wound infection

Data for wound infection (*Fig. 10e*) were reported by eight trials that recruited 1074 patients (*n* = 540 *versus n* = 534 in the three- and four-port groups, respectively). There was no statistically significant difference in the rate of wound infection between the three- and four-port groups (RR 1.24, 95 per cent c.i. 0.71 to 2.15; *P* = 0.46). A low level of heterogeneity was observed (*I^2^* = 0 per cent, χ^2^ = 2.11; *P* = 0.95).

#### Cosmetic satisfaction

Data for cosmetic satisfaction (*Fig. 10f*) were reported by three trials that recruited 205 patients (*n* = 101 *versus n* = 104 in the three- and four-port groups, respectively). Meta-analysis was performed on studies which analysed cosmetic satisfaction with a continuous scale at 7 days postoperatively. There was no statistically significant difference in cosmetic satisfaction between the three- and four-port groups (s.m.d. 0.08, 95 per cent c.i. −0.22 to 0.39; *P* = 0.59). A low level of heterogeneity was observed (*I^2^* = 15 per cent, χ^2^ = 2.35; *P* = 0.31).

#### Mortality

Mortality was reported by seven trials that recruited 890 patients (*n* = 450 *versus n* = 440 in the three- and four-port groups, respectively). There were no mortalities in either group.

#### Bile duct injury

Bile duct injury was reported by 13 trials that recruited 1631 patients (*n* = 819 *versus n* = 812 in the three- and four-port groups, respectively). There was one bile duct injury in the four-port group.

### Sensitivity analysis

Additional sensitivity analyses were performed for outcomes suitable for meta-analysis. Removing one study at a time, use of a fixed-effects model instead of a random-effects model, use of the m.d. instead of the s.m.d. (and vice versa) and use of an odds ratio instead of a RR did not change the statistical significance of any of our outcomes except for ‘spillage of biliary contents’, where the result became statistically significant and favoured the four-port group. Furthermore, a sensitivity analysis by excluding those trials in which the standard deviation was imputed or calculated did not change the statistical significance for any of our outcomes.

### Subgroup analysis

From our protocol, two subgroup analyses were planned but could not be performed. It was not possible to explore high risk of bias *versus* low risk of bias because the risk of bias analysis did not reveal any trials with low risk of bias. It was not possible to explore emergency *versus* elective cholecystectomy because no study provided data on the inclusion of an emergency laparoscopic cholecystectomy or ‘hot gallbladder’ operation.

### Summary-of-findings table

**Fig. 11:**
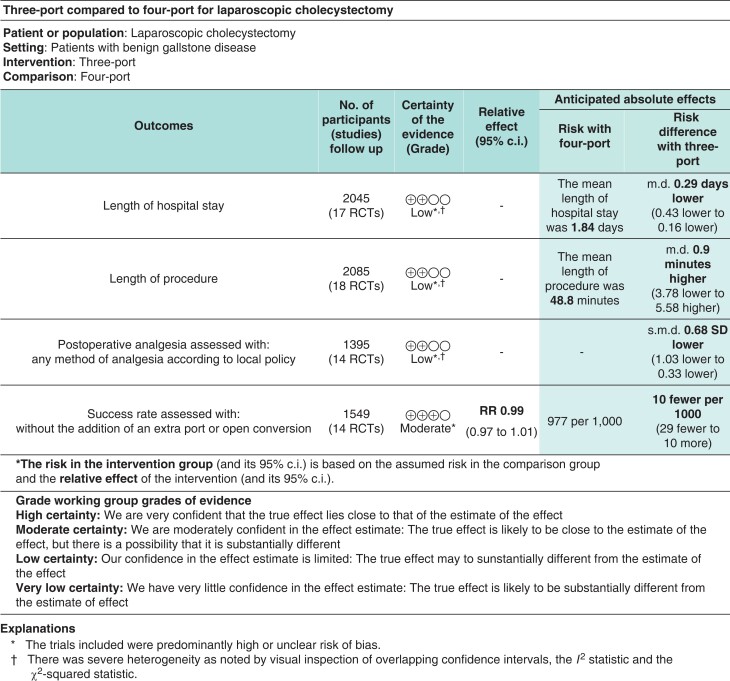
Summary of findings table for our primary outcomes

Primary outcomes were assessed according to the GRADE criteria, including length of hospital stay, length of procedure, and postoperative requirement of analgesia (low quality due to high risk of bias and high heterogeneity). The success rate was rated as moderate quality as the heterogeneity was low but risk of bias remained high. Overall, the quality of this evidence is low (*Fig. 11*).

GRADE levels of evidence, reasons for downgrading the level of evidence, and anticipated absolute effects. m.d., mean difference; s.m.d., standardized mean difference; RR, risk ratio.

## Discussion

The analysis of our primary outcomes suggests that the three-port approach is associated with a lower length of hospital stay and a reduction in postoperative analgesia requirement *versus* the four-port approach. There were no differences between the length of procedure and success rates between the two techniques. A possible explanation for the lower length of hospital stay and postoperative analgesia requirement could be due to a reduction in pain from less incisions.

However, a reduction in length of hospital stay may not be clinically significant if patients are operated on a day case list and discharged the same day. Elective day case laparoscopic cholecystectomy is now performed in the majority of patients, but more than half of our included studies were published before 2015, when this was less common^15^. Therefore, the results may only be applicable to patients who require at least an overnight stay. The proportion of patients discharged as day cases is probably a more meaningful outcome for future studies.

There was no statistically significant difference between the length of procedure and success rates between the two groups. This is probably because the three-port approach can generate equally good views without compromising safety. In addition, the time needed to insert a fourth port or close an extra incision probably had a minimal effect on total operating time. Experience level with each technique was felt to be subjective and was not measured in this review.

For secondary outcomes, there was no significant difference in the occurrence of adverse events, including gallbladder perforation, spillage of biliary contents, liver bed bleeding, wound infection, and cosmetic satisfaction. There was a moderate reduction in pain scores at 24 hours for the three-port group, in line with the reduction found in postoperative analgesia requirement. However, we are unable to draw any conclusions about pain scores beyond 24 hours. There was no difference in cosmetic satisfaction at 7 days, but any result is unlikely to be clinically significant as the scar has not fully remodelled within this time frame.

There were no mortalities (0 per cent) and only one bile duct injury (0.1 per cent) across the measured cohort. Rates of 30-day mortality and bile duct injury were 0.1 per cent and 0.3 per cent, respectively, from the Swedish Gallriks and SALT databases^16,17^. Given that mortality was only measured in 890 patients, it is unlikely that our sample size was adequately powered to measure differences in mortality between the two groups. The observed bile duct injury rate was lower than that measured by the SALT database. This is unsurprising given that the included studies randomized patients of relatively low age and low anaesthetic risk, and were operated on electively.

This systematic review proved to be robust for all outcomes subjected to sensitivity analysis except for spillage of biliary contents. This was the only outcome where there was a change in conclusion. It is possible that the four-port technique causes less spillage of biliary contents rather than there being no difference between the two groups.

There are two previous existing systematic reviews in the literature that examined three-ports *versus* four-ports for laparoscopic cholecystectomy. In 2009, Sun *et al*. performed a review with similar outcomes to our primary outcomes^18^. Five trials were included in their study, and they concluded that there was no statistically significant difference in any of their outcomes but did not derive any recommendations from them. Gurusamy *et al*. looked at fewer than four ports *versus* four ports^19^. Overall, they concluded there was very low-quality evidence and that it was insufficient to determine whether there was any clinical benefit in using fewer than four ports.

The differences observed between this study and that of Sun *et al*.^18^ essentially reflect a more up-to-date synthesis of the available literature. Four of their five studies were included in this systematic review, but we also included more recent RCTs. The study of Sun *et al*.^18^ included one non-English text (written in Mandarin), which was an exclusion criterion in our study. However, it is unlikely that this study, which randomized 96 participants, would affect the results^20^.

The differences observed between this review and that of Gurusamy *et al*.^19^ could be due to the fact that seven of nine of their included studies looked at single-incision *versus* four-port laparoscopic cholecystectomy. Only two of their included studies randomized participants to three-port *versus* four-port^21,22^. Also, their definition for a four-port control group was strictly defined as two 10-mm ports and two 5-mm ports; otherwise, studies were excluded. This study included studies with four ports of all sizes as long as the location of the ports adhered to conventional port placement. The implications of these results could be more generalizable given the variance in port size found in normal clinical practice.

This review has limitations. Firstly, we imputed the standard deviation of some studies based on other studies we felt had similar sample sizes and inclusion/exclusion criteria. We were unable to perform a subgroup analysis of high risk of bias *versus* low risk of bias trials and patients operated on in an emergency setting *versus* those being electively operated on. We were therefore unable to explore the high level of heterogeneity found in some of our primary outcomes. We were not able to the measure experience level of the surgeons in an objective way. We felt that a questionnaire asking about years of training or number of cholecystectomies performed would result in reporting bias and therefore we did not do this. Using the standardized GRADE approach, the quality of this evidence is low.

This review suggests that the three-port method is associated with a reduction in length of hospital stay and postoperative analgesia requirement with otherwise comparable outcomes. The decision to use three port to achieve a shorter length of hospital stay or reduced requirement for postoperative analgesia should not be at the expense of safe dissection of Calot’s triangle.

## Supplementary Material

zrac013_Supplementary_Data

## Data Availability

The authors declare that the data, methods, and materials used to conduct the research are made available in the main body of the manuscript and supplementary material attached.
