# Supplementary material for: Three-port versus four-port technique for laparoscopic cholecystectomy: systematic review and meta-analysis
Source: BJS Open. 2022 Mar 31;6(2):zrac013. doi: 10.1093/bjsopen/zrac013 (PMC8969828; doi:10.1093/bjsopen/zrac013)
Supplement: zrac013_Supplementary_Data [file zrac013_supplementary_data.zip › Supplementary_Appendix_2.docx]

Appendix S2

Search No. Search strategy

#1 (Three-port).ti,ab

#2 (Three-ports).ti,ab

#3 (Three-trocar).ti,ab

#4 (Three-trocars).ti,ab

#5 (3-port).ti,ab

#6 (3-ports).ti,ab

#7 (3-trocar).ti,ab

#8 (3-trocars).ti,ab

#9 (Three ADJ2 port).ti,ab

#10 (Three ADJ2 ports).ti,ab

#11 (Three ADJ2 trocar).ti,ab

#12 (Three ADJ2 trocars).ti,ab

#13 (3 ADJ2 port).ti,ab

#14 (3 ADJ2 ports).ti,ab

#15 (3 ADJ2 trocar).ti,ab

#16 (3 ADJ2 trocars).ti,ab

#17 #1 OR #2 OR … OR #15 OR #16

#18 (Four-port).ti,ab

#19 (Four-ports).ti,ab

#20 (Four-trocar).ti,ab

#21 (Four-trocars).ti,ab

#22 (4-port).ti,ab

#23 (4-ports).ti,ab

#24 (4-trocar).ti,ab

#25 (4-trocars).ti,ab

#26 (Four ADJ2 port).ti,ab

#27 (Four ADJ2 ports).ti,ab

#28 (Four ADJ2 trocar).ti,ab

#29 (Four ADJ2 trocars).ti,ab

#30 (4 ADJ2 port).ti,ab

#31 (4 ADJ2 ports).ti,ab

#32 (4 ADJ2 trocar).ti,ab

#33 (4 ADJ2 trocars).ti,ab

#34 #18 OR #19 OR … OR #32 OR #33

#35 (Laparoscopic cholecystectomy[MeSH Terms]).explode all trees

#36 (Laparoscopic cholecystectomy).ti,ab

#37 (Gallbladder removal).ti,ab

#38 (Removal ADJ2 gallbladder).ti,ab

#39 #35 OR #36 OR #37 OR #38

#40 #17 AND #34 AND #39
