# Supplementary material for: Three-port versus four-port technique for laparoscopic cholecystectomy: systematic review and meta-analysis
Source: BJS Open. 2022 Mar 31;6(2):zrac013. doi: 10.1093/bjsopen/zrac013 (PMC8969828; doi:10.1093/bjsopen/zrac013)
Supplement: zrac013_Supplementary_Data [file zrac013_supplementary_data.zip › Supplementary_Appendix_3.docx]

References to Included Studies

**Agarwal** KA, Kamal R. Evaluation of efficacy of three-port v/s standard four-port laparoscopic cholecystectomy in patients of symptomatic cholelithiasis: an institutional based study. Int J Med Res Prof. Jan 2018;4(1):658-60.

**Bari** SU, Islam FU, Rather AA, et al. Three port versus four port laparoscopic cholecystectomy: a prospective comparative clinical study. Int J Res Med Sci. Aug 2019;7(8):3054-59.

**Cerci** C, Tarhan OR, Barut I, et al. Three-port versus four-port laparoscopic cholecystectomy. Hepatogastroenterol. 2007;54:15-16.

**Eroler** E, Dilektasli E, Tihan D, et al. Reducing one port in laparoscopic cholecystectomy: does that really make a difference? Int J Clin Exp Med. 2016;9(6):11558-65.

**Gupta** A, Shrivastava UK, Kumar P, et al. Minilaparoscopic versus laparoscopic cholecystectomy: a randomised controlled trial. Trop Gastroenterol. 2005;26:149-51.

**Harsha** HS, Gunjiganvi M, Singh CA, et al. A study of three-port versus four-port laparoscopic cholecystectomy. J Med Soc. 2013;27(3):208-11.

**Khorgami** Z, Shoar S, Anbara T, et al. A randomized clinical trial comparing 4-port, 3-port and single-incision laparoscopic cholecystectomy. J Invest Surg. 2014;27(3):147-54.

**Kumar** M, Agrawal CS, Gupta RK. Three-port versus standard four-port laparoscopic cholecystectomy: a randomized controlled clinical trial in a community-based teaching hospital in eastern Nepal. JSLS. 2007;11:358-62.

**Liu** E, Li Z, Wang N, et al. A prospective, randomized, controlled trial of three-port laparoscopic cholecystectomy versus conventional four-port laparoscopic cholecystectomy: is the fourth port really required? Int J Clin Exp Med. 2016;9(2):3055-61.

**Mohamed** AAEA, Zaazou MMT. Three-port versus conventional four-port laparoscopic cholecystectomy: a comparative study. Egypt J Surg. 2020;39:119-23.

**Moran** M, Ozmen MM, Bilgiç I, et al. Is the number of trocars important in laparoscopic cholecystectomy? Eur J Endosc Laparosc Surg. 2014;1:24-29.

**Reshie** TA, Rather ZM, Bhat MY, et al. Three port versus four port laparoscopic cholecystectomy: a comparative study. Int J Adv Res. 2015;3(10):1040-44.

**Shah** SF, Waqar SH, Chaudry MA, et al. Three ports versus four ports laparoscopic cholecystectomy. Rawal Med J. 2017;42(3):359-62.

**Sharma** PK, Mehta KS. Three port versus standard four port laparoscopic cholecystectomy – a prospective study. JK Sci. 2015;17(1):38-42.

**Singal** R, Goyal P, Zaman M, et al. Comparison of three-port vs four-port laparoscopic cholecystectomy in a medical college in the periphery. World J Laparosc Surg. 2017;10(1):12-16.

**Singhal** P, Aggarwal A, Altamash S, et al. A comparative evaluation of three port versus standard four port laparoscopic cholecystectomy in SGRRIMHS and SMIH Dehradun. Int Surg J. Aug 2019;6(8):2900-04.

**Trichak** S. Three-port vs standard four-port laparoscopic cholecystectomy. Surg Endosc. 2003;17:1434-36.

**Vejdan** SAK, Khosravi M, Amirian Z, et al. Comparison of 3-port with standard 4-port laparoscopic cholecystectomy: a clinical trial. J Surg Trauma. 2020;8(2):52-57.
