# Supplementary material for: Three-port versus four-port technique for laparoscopic cholecystectomy: systematic review and meta-analysis
Source: BJS Open. 2022 Mar 31;6(2):zrac013. doi: 10.1093/bjsopen/zrac013 (PMC8969828; doi:10.1093/bjsopen/zrac013)
Supplement: zrac013_Supplementary_Data [file zrac013_supplementary_data.zip › Supplementary_Appendix_4.docx]

Appendix S4

Randomisation: We deemed a study to be low risk of bias if the sequence generation was likely to be truly random such as computer generated or table of random numbers. If insufficient information was provided then we classed this as unclear risk of bias. A study was high risk of bias if the method used was quasi-randomisation or if the baseline demographics suggest that the two groups were distinctly different.

Allocation concealment: We deemed a study to be low risk of bias if the method of allocation concealment meant that the group the participant was allocated to was likely to be unpredictable e.g. central allocation or sealed envelopes. If insufficient information was provided then we classed this as unclear risk of bias. A study was high risk of bias if participants or personnel could know in advance which group a patient was allocated to before they receive the intervention e.g. open list.

Blinding of participants: Although postoperatively it is likely that a patient may deduce which procedure they underwent, we deemed a study to be low risk of bias if measures were taken as much as possible to blind the patient e.g. four dressings at the operative sites even if three-ports were used. If insufficient information was provided then we classed this as unclear risk of bias. A study was high risk of bias if there was no blinding and the outcomes were likely to be affected by a lack of blinding.

Blinding of outcome assessors: Whilst it was impossible to blind the operating surgeon, we deemed a study to be low risk of bias if an independent outcome assessor was responsible for collecting the data, such as a blinded nurse administering analgesia or a separate surgeon responsible for decisions regarding discharge. For this domain, we did not distinguish between unclear and high risk of bias. Studies were classed as high risk of bias if insufficient information was provided since it was likely that any knowledge of which intervention the patient had received would influence the outcome.

Missing outcome data: We deemed a study to be low risk of bias if there were no post-randomisation dropouts or the reason for dropout was given and an intention-to-treat analysis was performed. If insufficient information was provided then we classed this as unclear risk of bias. A study was high risk of bias if outcomes suggested missing information without giving reasons or a per-protocol analysis was performed.

Selective reporting: We deemed a study to be low risk of bias if the trial protocol was available and all pre-specified outcomes were reported. If the trial protocol was not available, we still deemed the trial to be low risk of bias if morbidity outcomes including complications were individually reported. If morbidity outcomes including complications were not provided, then we classed this as unclear risk of bias. Trials were high risk of bias if they clearly deviated from the trial protocol with non-reporting of certain outcomes. In addition, we classed a trial as high risk of bias if there were claims of significance or non-significance without providing the raw data.

Agarwal

| Bias | Authors’ Judgement | Support for Judgement |
| --- | --- | --- |
| Random sequence generation  (selection bias) | Unclear risk | No information |
| Allocation concealment  (selection bias) | Unclear risk | No information |
| Blinding of participants and  personnel (performance bias) | Low risk | Four surgical tapes applied to standard 4-port sites in both  groups at the end of the operation. |
| Blinding of outcome  assessment (detection bias) | High risk | No information on who the assessor was, and assessment of  the outcome would likely be influenced by knowledge of the  intervention. |
| Incomplete outcome data  (attrition bias) | Low risk | No attrition. |
| Selective reporting (reporting  bias) | High risk | Multiple ways of measuring pain were used, but only VAS  and analgesic requirement were reported. Also, there were other outcomes (e.g. postoperative stay, patient satisfaction score on surgery and scars) that were reported as similar but no data were presented. |

Bari

| Bias | Authors’ Judgement | Support for Judgement |
| --- | --- | --- |
| Random sequence generation  (selection bias) | Unclear risk | No information |
| Allocation concealment  (selection bias) | Unclear risk | No information |
| Blinding of participants and  personnel (performance bias) | Unclear risk | No information |
| Blinding of outcome  assessment (detection bias) | Low risk | An independent doctor assessed the pain score. |
| Incomplete outcome data  (attrition bias) | Low risk | No attrition. |
| Selective reporting (reporting  bias) | Low risk | Morbidity outcomes individually reported. |

Cerci

| Bias | Authors’ Judgement | Support for Judgement |
| --- | --- | --- |
| Random sequence generation  (selection bias) | Unclear risk | No information |
| Allocation concealment  (selection bias) | Unclear risk | No information |
| Blinding of participants and  personnel (performance bias) | Unclear risk | No information |
| Blinding of outcome  assessment (detection bias) | High risk | No information on who the assessor was, and assessment of  the outcome would likely be influenced by knowledge of the  intervention. |
| Incomplete outcome data  (attrition bias) | Low risk | No attrition. |
| Selective reporting (reporting  bias) | Unclear risk | Morbidity outcomes not individually reported. No trial protocol available. |

Eroler

| Bias | Authors’ Judgement | Support for Judgement |
| --- | --- | --- |
| Random sequence generation  (selection bias) | High risk | Imbalanced baseline demographic suggests a problem with the randomisation process. |
| Allocation concealment  (selection bias) | Unclear risk | Before the operation patients were blinded on the treatment  group that they were enrolled. No mention of concealment  from investigators. |
| Blinding of participants and  personnel (performance bias) | Low risk | Before the operation patients were blinded on the treatment  group that they were enrolled. |
| Blinding of outcome  assessment (detection bias) | Low risk | Pain score was self-reported by patients who were blinded to  the treatment group that they were enrolled. The other  outcomes were assessed by an unknown assessor but these  were objective measurements that did not require judgement. |
| Incomplete outcome data  (attrition bias) | High risk | 6 out of 30 in the three-port group were excluded due to  conversion to four-port technique; a further 6 participants were enrolled to replace them. Not intention to treat. |
| Selective reporting (reporting  bias) | Low risk | Morbidity outcomes individually reported. |

Gupta

| Bias | Authors’ Judgement | Support for Judgement |
| --- | --- | --- |
| Random sequence generation  (selection bias) | Low risk | 'Random selection of a sealed envelope by the patient.' |
| Allocation concealment  (selection bias) | Unclear risk | 'Random selection of a sealed envelope by the patient.' |
| Blinding of participants and  personnel (performance bias) | Unclear risk | No information |
| Blinding of outcome  assessment (detection bias) | Low risk | All pain assessments were collected by a blinded  observer. |
| Incomplete outcome data  (attrition bias) | Low risk | No attrition. |
| Selective reporting (reporting  bias) | Unclear risk | Morbidity outcomes not individually reported. No trial protocol available. |

Harsha

| Bias | Authors’ Judgement | Support for Judgement |
| --- | --- | --- |
| Random sequence generation  (selection bias) | Low risk | Patients were allocated by computer generated simple  random sampling method. |
| Allocation concealment  (selection bias) | Unclear risk | No information. |
| Blinding of participants and  personnel (performance bias) | Unclear risk | No information. |
| Blinding of outcome  assessment (detection bias) | High risk | No information on who the assessor was, and assessment of  the outcome would likely be influenced by knowledge of the  intervention. |
| Incomplete outcome data  (attrition bias) | Low risk | No attrition. |
| Selective reporting (reporting  bias) | Low risk | Morbidity outcomes not individually reported. |

Khorgami

| Bias | Authors’ Judgement | Support for Judgement |
| --- | --- | --- |
| Random sequence generation  (selection bias) | Low risk | Central randomisation process based on a computer-generated list coordinated by an independent statistician not otherwise involved in the study. |
| Allocation concealment  (selection bias) | Unclear risk | No information. |
| Blinding of participants and  personnel (performance bias) | Low risk | Four dressings placed over the usual sites to blind both the  patients and nurses responsible for gathering data. |
| Blinding of outcome  assessment (detection bias) | Low risk | Four dressings placed over the usual sites to blind both the  patients and nurses responsible for gathering data. |
| Incomplete outcome data  (attrition bias) | Low risk | No attrition. |
| Selective reporting (reporting  bias) | Low risk | All outcomes reported as per trial protocol. |

Kumar

| Bias | Authors’ Judgement | Support for Judgement |
| --- | --- | --- |
| Random sequence generation  (selection bias) | Unclear risk | No information. |
| Allocation concealment  (selection bias) | Unclear risk | No information. |
| Blinding of participants and  personnel (performance bias) | Low risk | All patients were blinded to the type of operation they underwent. |
| Blinding of outcome  assessment (detection bias) | Low risk | Assessor was an independent surgeon who was blind to  the type of surgery that the patients underwent. |
| Incomplete outcome data  (attrition bias) | Low risk | No attrition. |
| Selective reporting (reporting  bias) | Unclear risk | Morbidity outcomes not individually reported. |

Liu

| Bias | Authors’ Judgement | Support for Judgement |
| --- | --- | --- |
| Random sequence generation  (selection bias) | Low risk | Each subject was given a random number, and they were  ranked numerically according to the assigned random number. Odd ranks were assigned to group A (three-port), even ranks to group B (four-port). |
| Allocation concealment  (selection bias) | Unclear risk | No information. |
| Blinding of participants and  personnel (performance bias) | Unclear risk | No information. |
| Blinding of outcome  assessment (detection bias) | Low risk | Assessor was blinded. |
| Incomplete outcome data  (attrition bias) | High risk | Per protocol analysis. 245 recruited but 216 analysed due to lost to follow up |
| Selective reporting (reporting  bias) | High risk | During the operation the three-port LC could be changed to a  four-port LC if needed. No mention of how many were  changed to four-port. |

Mohamed

| Bias | Authors’ Judgement | Support for Judgement |
| --- | --- | --- |
| Random sequence generation  (selection bias) | Unclear risk | No information. |
| Allocation concealment  (selection bias) | Unclear risk | No information. |
| Blinding of participants and  personnel (performance bias) | Unclear risk | There is mention of the use of surgical adhesive tapes on  port sites, but unclear as to the number of tapes applied on  each patient. |
| Blinding of outcome  assessment (detection bias) | High risk | No information on who the assessor was, and assessment of  the outcome would likely be influenced by knowledge of the  intervention. |
| Incomplete outcome data  (attrition bias) | Unclear risk | There is some conversion to four ports or even open surgery;  no information as to how the missing data is handled. |
| Selective reporting (reporting  bias) | Low risk | Most outcomes reported ('operative difficulty' was not  reported although was specified as one of their outcomes;  may have been considered synonymous with 'operative  time'). |

Moran

| Bias | Authors’ Judgement | Support for Judgement |
| --- | --- | --- |
| Random sequence generation  (selection bias) | Low risk | Patients were randomised by the clinic secretary with  protocol numbers |
| Allocation concealment  (selection bias) | Unclear risk | No information. |
| Blinding of participants and  personnel (performance bias) | Unclear risk | No information. |
| Blinding of outcome  assessment (detection bias) | Low risk | An independent physcian unaware of the procedures  assessed the pain score and patient satisfaction scores |
| Incomplete outcome data  (attrition bias) | Low risk | No attrition. |
| Selective reporting (reporting  bias) | Low risk | Morbidity outcomes individually reported. |

Reshie

| Bias | Authors’ Judgement | Support for Judgement |
| --- | --- | --- |
| Random sequence generation  (selection bias) | Unclear risk | No information. |
| Allocation concealment  (selection bias) | Unclear risk | No information. |
| Blinding of participants and  personnel (performance bias) | Unclear risk | No information. |
| Blinding of outcome  assessment (detection bias) | High risk | No information on who the assessor was, and assessment of  the outcome would likely be influenced by knowledge of the  intervention. |
| Incomplete outcome data  (attrition bias) | High risk | Different sample sizes were used for different outcomes with no clear reasons given. |
| Selective reporting (reporting  bias) | Low risk | Morbidity outcomes individually reported. |

Shah

| Bias | Authors’ Judgement | Support for Judgement |
| --- | --- | --- |
| Random sequence generation  (selection bias) | Low risk | Randomised by a computer-generated table of random  numbers. |
| Allocation concealment  (selection bias) | Unclear risk | No information. |
| Blinding of participants and  personnel (performance bias) | Unclear risk | No information. |
| Blinding of outcome  assessment (detection bias) | High risk | No information on who the assessor was, and assessment of  the outcome would likely be influenced by knowledge of the  intervention. |
| Incomplete outcome data  (attrition bias) | Low risk | No attrition. |
| Selective reporting (reporting  bias) | Low risk | Morbidity outcomes individually reported. |

Sharma

| Bias | Authors’ Judgement | Support for Judgement |
| --- | --- | --- |
| Random sequence generation  (selection bias) | Unclear risk | No information. |
| Allocation concealment  (selection bias) | Unclear risk | No information. |
| Blinding of participants and  personnel (performance bias) | Unclear risk | No information. |
| Blinding of outcome  assessment (detection bias) | High risk | No information on who the assessor was, and assessment of  the outcome would likely be influenced by knowledge of the  intervention. |
| Incomplete outcome data  (attrition bias) | Low risk | No attrition. |
| Selective reporting (reporting  bias) | Low risk | Morbidity outcomes individually reported. |

Singal

| Bias | Authors’ Judgement | Support for Judgement |
| --- | --- | --- |
| Random sequence generation  (selection bias) | Unclear risk | No information. |
| Allocation concealment  (selection bias) | Unclear risk | No information. |
| Blinding of participants and  personnel (performance bias) | Unclear risk | No information. |
| Blinding of outcome  assessment (detection bias) | High risk | No information on who the assessor was, and assessment of  the outcome would likely be influenced by knowledge of the  intervention. |
| Incomplete outcome data  (attrition bias) | High risk | Different sample sizes were used for different outcomes with no clear reasons given. |
| Selective reporting (reporting  bias) | High risk | Outcomes were unclear and claims of significance without p  values. |

Singhal

| Bias | Authors’ Judgement | Support for Judgement |
| --- | --- | --- |
| Random sequence generation  (selection bias) | Unclear risk | No information. |
| Allocation concealment  (selection bias) | Unclear risk | No information. |
| Blinding of participants and  personnel (performance bias) | Unclear risk | No information. |
| Blinding of outcome  assessment (detection bias) | High risk | No information on who the assessor was, and assessment of  the outcome would likely be influenced by knowledge of the  intervention. |
| Incomplete outcome data  (attrition bias) | Low risk | No attrition. |
| Selective reporting (reporting  bias) | Low risk | Morbidity outcomes individually reported. |

Trichak

| Bias | Authors’ Judgement | Support for Judgement |
| --- | --- | --- |
| Random sequence generation  (selection bias) | Unclear risk | No information. |
| Allocation concealment  (selection bias) | Unclear risk | No information. |
| Blinding of participants and  personnel (performance bias) | Unclear risk | No information. |
| Blinding of outcome  assessment (detection bias) | High risk | No information on who the assessor was, and assessment of  the outcome would likely be influenced by knowledge of the  intervention. |
| Incomplete outcome data  (attrition bias) | Low risk | No attrition. |
| Selective reporting (reporting  bias) | Unclear risk | Morbidity outcomes not individually reported. |

Vejdan

| Bias | Authors’ Judgement | Support for Judgement |
| --- | --- | --- |
| Random sequence generation  (selection bias) | Low risk | Balanced block randomisation used. |
| Allocation concealment  (selection bias) | Unclear risk | No information. |
| Blinding of participants and  personnel (performance bias) | Unclear risk | Reported as double-blind study, but no further information provided. |
| Blinding of outcome  assessment (detection bias) | Low risk | The data were collected by a nursing expert who was  blinded to the patients' study groups. |
| Incomplete outcome data  (attrition bias) | Low risk | No attrition. |
| Selective reporting (reporting  bias) | Unclear risk | Morbidity outcomes not individually reported. |
